# Health insurance enrollment and maternal health service utilization using Ghana Demographic and Health Survey, 2022

**DOI:** 10.1371/journal.pone.0325240

**Published:** 2025-06-26

**Authors:** Kennedy Mensah Osei, Danik Iga Prasiska, Durga Datta Chapagain, Vasuki Rajaguru, Sun Joo Kang, Tae Hyun Kim, Sang Gyu Lee, Whiejong Han

**Affiliations:** 1 Global Health Security, Graduate School of Public Health, Yonsei University, Seoul, South Korea; 2 Tamale Teaching Hospital, Ministry of Health, Tamale, Northern Region, Ghana; 3 Healthcare Management, Graduate School of Public Health, Yonsei University, Seoul, South Korea; 4 Department of Global Health and Disease Control, Graduate School of Public Health, Yonsei University, Seoul, South Korea; 5 Department of Preventive Medicine, College of Medicine, Yonsei University, Seoul, South Korea; Nurses' and Midwives Training College, GHANA

## Abstract

**Background:**

Maternal health services utilization is essential in the reduction of maternal mortality. Despite the implementation of a national health insurance scheme in 2003, Ghana still reports universal health coverage service index below the global average. This study investigates the association between health insurance coverage and maternal health service utilization.

**Methods:**

This study utilized data from the Ghana Demographic and Health Survey (GDHS) conducted in 2022. The independent variable of the study was health insurance coverage, and the outcome variable was maternal health service utilization by assessing indicators including the timing of the first ANC visit, completing the recommended number of ANC visits, skilled birth attendance, facility-based delivery, and post-natal care. The data was analyzed for both descriptive statistics and logistic regression.

**Results:**

The study sample consisted of 4303 women of reproductive age who had live births within the past 5 years of the survey. Health insurance coverage is associated with the likely odds of post-natal care (aOR 1.56; 1.15–2.12). Counterintuitively, women who were insured were less likely to give birth in a health facility (aOR 0.59; 0.45–0.78) in the presence of a skilled birth attendant (aOR 0.70; 0.57–0.86).

**Conclusion:**

This study shows that while health insurance coverage can boost maternal health service utilization, the implementation mechanisms of these policies play a more critical role. Addressing challenges like out-of-pocket payments for insured individuals is essential to enhance service utilization under the policy.

## Background

Global maternal and child mortality has declined significantly over the past decades due to Millennium Development Goals (MDGs) and Sustainable Development Goals (SDGs) policies and programs, as well as national and international efforts [1,2]. SDG target 3.1 aims to reduce maternal mortality to less than 70 maternal deaths per 100,000 live births by 2030 [3]. However, as of 2020, the global maternal mortality ratio (MMR) was estimated to be 223 maternal deaths per 100,000 live births with sub-Saharan Africa accounting for a higher share of global maternal deaths with about 69% [2]. Ghana reported an MMR of 310 maternal deaths per 100,000 live births in 2022 [4].

SDG target 3.8 aims to achieve Universal Health Coverage (UHC) including financial risk protection, healthcare services access, and safe, effective, quality, and affordable essential medicines and vaccines for all [3]. Despite the introduction of a National Health Insurance Scheme (NHIS) in 2003, Ghana recorded a UHC service coverage index of 48% well below the global average of 68% as reported by the World Health Organization (WHO) [5,6]. The NHIS provides free maternal health services for all pregnant women at both public and accredited private health facilities provided the women are registered with the scheme and hold a valid NHIS card at the point of service [7,8]. The premium and processing fees required for registering and obtaining a valid NHIS card sometimes become a barrier to accessing this ostensibly free maternal health services. This exposes a substantial portion of the population to financial risk in accessing essential health services including maternal healthcare services.

Improvement in maternal healthcare services through the provision of antenatal care, skilled birth attendance, facility-based delivery, and post-natal care are essential in the reduction of maternal and child mortality [9]. However, several barriers hinder the optimal utilization of maternal healthcare services especially in low-middle-income countries (LMICs). Healthcare cost is a fundamental barrier to the optimal utilization of essential health services in most LMICs. Previous studies in other low middle-income countries have associated health insurance coverage (HIC) with essential health services utilization including maternal health services [10–13].

This study explores the association between health insurance coverage and maternal health services utilization based on Andersen’s Health Care Utilization Model [14]. The model espouses three factors associated with healthcare utilization: social and demographic factors, enabling factors and individuals’ perceptions of their health.

There is considerable evidence on the association of health services utilization and health insurance coverage globally, however, few studies have specifically dealt with this association in West Africa and specifically Ghana. The need for further studies in Ghana is bolstered by the relatively high maternal mortality rate even though the NHIS was implemented some two decades ago. Hence this study seeks to fill the gap in the literature to assess the association between health insurance coverage and maternal health service utilization by analyzing the most recently available 2022 Demographic and Health Survey (DHS) data.

## Methods

### Data source

This study utilized data from the Ghana Demographic and Health Survey (GDHS) conducted in 2022 by the Ghana Statistical Service in collaboration with the Ministry of Health. The Demographic and Health Survey, which is conducted in over 80 low- and middle-income countries is a nationally representative survey that collects data on health indicators including maternal health service utilization. The survey employs a two-stage sampling method for data collection using a standardized questionnaire [15].A total of 4,303 women of childbearing age (15–49 years), who had given birth within the five years preceding the survey, were included in this study. The data were obtained from the Individual Women’s Data (Individual Recode – IR) file, which includes one record for every eligible woman as identified in the household schedule. This dataset contains all information collected through the women’s questionnaire, along with selected variables from the household questionnaire. The variables used is this study is defined in **Table S3** in the supplementary document...

### Ethical considerations

The required data for this study was obtained from the official DHS website: http://www.dhsprogram.com on request by email. The protocol for DHS surveys is approved by the Ethics Committee of ORC Macro Inc. This study utilized anonymized secondary data available in the public domain of DHS. The DHS program explicitly seeks the consent of survey respondents. However, the authors obtained approval from DHS for the reuse of the data. This study adheres to the STROBE guidelines for reporting observational studies.

### Variables

#### Outcome variables.

This study examines five indicators of maternal health service utilization based on the WHO and Ghana Health Service (GHS) recommended guidelines on essential MHC services including the timing of the first Antenatal care (ANC) visit, completion of the recommended number of ANC visit, facility-based delivery and post-natal Care (PNC). A first ANC visit within the first trimester of pregnancy is recommended for pregnant women. A minimum of four ANC visits is the recommended number of ANC visits, giving birth at a healthcare facility under the supervision and care of a qualified healthcare worker is recommended by the guideline.

#### Independent variable of interest.

The primary variable of interest in this study was the respondent’s enrollment in the national health insurance scheme using the question “Are you registered with the National Health Insurance Scheme” and the response was considered as “Yes” and “No” for insured and non-insured respondents respectively.

#### Covariates.

To account for confounding in the analysis, this study controlled for maternal age groups which were categorized into four groups: 15–24, 25–34, 35–42, and 42–49. Marital status was classified as either single or married. The education level was categorized into four groups: primary, secondary, higher, and no education. Employment (employed and unemployed) and residence (urban and rural) were segmented into two categories. The region of residence was defined as one of 16 administrative regions. Parity was categorized into 3 groups (3 children or less, more than 3 but less than 6 children and more than 6 children) and religion was divided into 4 categories (no religion, Christianity, Islam and Traditional religion). Respondents were classified into wealth index (lowest, second, middle fourth, and highest), and exposure to the internet was defined by 4 categories (not at all, less than once a week, at least once a week, and almost every day). Wealth index and region of residence were employed to determine inequalities in access to Maternal health services. In our analysis, we included covariates that have been identified as significant predictors in previous research. Specifically, these studies have demonstrated that variables such as age, education level, wealth index, religion, and region of residence are crucial for accurately modeling the relationship between health insurance enrolment and maternal health service utilization. By incorporating these covariates, we aim to control potential confounding factors and ensure that our findings are robust and comparable to existing literature [16–20].

### Data analysis

Our study analyzed data using both descriptive and regression model methods with SAS 9.4. Primary analyses to determine the distribution of respondents’ socio-demographic characteristics and Health insurance coverage by MHC services utilization were performed. Further analysis was done to determine the predictors of health insurance coverage among women of childbearing age. The analysis focused on MHC services utilization, including the timing of the first ANC visit, completion of the 4 or more recommended number of ANC visits, childbirth with skilled labor attendance, facility-based delivery, and post-natal care. A significance level of p < 0.05 (two-tailed) was used for all analyses. A multivariate logistic regression was done to determine the association between HIC and maternal health services utilization indicators. The adjusted odds ratios (aOR) were calculated to assess the strength of the associations, and 95% confidence intervals (CIs) were used to test for statistical significance. To assess the presence of multicollinearity among the independent variables, we calculated the Variance Inflation Factor (VIF) for each predictor in the regression model. The Variance Inflation Factor (VIF) values for the independent variables in the model ranged from 1.028 to 2.121, with a mean VIF of 1.44. The standard error ranges from 0.00032 to 0.001174 for all independent variables. These values suggest that there is no significant multicollinearity, indicating that the independent variables are not correlated. Details of the VIF results are shown in the supplementary document Table S1.

## Results

### Health insurance coverage and utilization of maternal healthcare services among women of reproductive age

The study sample consisted of 4303 women of reproductive age who had live births within the past 5 years of the survey. Among all the respondents, 76.74% indicated health insurance coverage. Age, education level, residence, employment status, religion, wealth quintile, parity, media exposure(internet), and region of residence were determined to have an association with health insurance coverage. (Table 1). A total of 2923 respondents reported their first ANC visit within the first trimester of pregnancy representing 67.93% of the sample size while 1380 did not report their first ANC visit within the recommended first trimester of their pregnancy. For at least 4 ANC visits during pregnancy, 92.77% of the respondents reported having completed 4 or more ANC visits. A similarly high proportion (81.66%) of the respondents reported having a skilled birth attendant during childbirth compared with 18.34% who did not have a skilled birth attendant present during childbirth. The proportion of the respondents reporting facility-based delivery and post-natal care was 89.98% and 94.93% respectively as shown in Table S2.

**Table 1:** Distribution of study respondents across health insurance coverage (N = 4303).

	Health Insurance Coverage				*p*-value
Variables	Yes		No		
	n	%	n	%	
Total	3302	76.74	1001	23.26	
**Age**					*
15-24	1042	31.56	314	31.37	
25-34	1470	44.52	400	39.96	
35-42	686	20.78	249	24.88	
42-49	104	3.15	38	3.8	
**Marital status**					
Single	457	13.84	157	15.68	
Married	2845	86.16	844	84.32	
**Education**					***
No education	790	23.92	305	30.47	
Primary	469	14.2	180	17.98	
Secondary	1685	51.03	487	48.65	
Higher	358	10.84	29	2.9	
**Residence**					*
Urban	1556	47.12	422	42.16	
Rural	1746	52.88	579	57.84	
**Employment**					**
Unemployed	829	25.11	177	17.68	
Employed	2473	74.89	824	82.32	*
**Religion**					
No religion	40	1.21	31	3.1	
Christianity	2121	64.23	634	36.34	
Islam	1107	33.53	308	30.77	
Traditional religion	34	1.03	28	2.8	
**Wealth quintile**					***
Lowest	833	25.23	327	32.67	
Second	808	24.47	251	25.07	
Middle	625	18.93	213	21.28	
Fourth	569	17.23	132	13.19	
Highest	467	14.14	78	7.79	
**Parity**					***
Less than 4	2229	67.5	605	60.44	
More than 3 less than 6	723	21.9	248	24.78	
More than 5	350	10.6	148	14.79	
**Internet exposure**					***
Not at all	2223	67.32	787	78.62	
Less than once a week	113	3.42	37	3.7	
At least once a week	315	9.54	69	6.89	
Almost everyday	651	19.72	108	10.79	
**Region**					***
Western	131	3.97	58	5.79	
Central	155	4.69	65	6.49	
Greater Accra	166	5.03	51	5.09	
Volta	140	4.24	45	4.5	
Eastern	182	5.51	19	1.9	
Ashanti	219	6.63	63	6.29	
Western North	163	4.94	36	3.6	
Ahafo	194	5.88	51	5.09	
Bono	162	4.91	41	4.1	
Bono East	232	7.03	62	6.19	
Oti	185	5.6	52	5.19	
Northern	287	8.69	91	9.09	
Savannah	210	6.36	119	11.89	
North East	304	9.21	92	9.19	
Upper East	271	8.21	73	7.29	
Upper West	241	7.3	68	6.79	

*p *< 0.05, *p* < 0.01 and *p *< 0.001 indicated by *, ** and *** respectively.

### Distribution of maternal health utilization across health insurance and other socio-demographic characteristics of participants

The characteristics of the 4303 respondents, categorized according to utilization of maternal health services are shown in Table 2. Assessment of maternal health utilization services across health insurance coverage revealed a statistically significant proportion of respondents utilize maternal health services when they are insured. Among respondents in this study, for those found to have accessed ANC in the first trimester of their pregnancy, 68.78% were insured while 65.13% of those who were not insured with a statistically significant *p*-value of < 0.05. Statistically similar results were found in other maternal health utilization services; completing the recommended number of ANC visits was recorded 93.22% of insured respondents versus 91.31% of uninsured respondents (*p*-value < 0.05),. Finally, there was observed a statistically significant association between timing of first ANC visit (*p *< 0.001), completion of recommended ANC visits (*p *< 0.001), skilled birth attendance (*p *< 0.001), and post-natal care (*p *< 0.01), across different wealth quintiles. Paradoxically, a higher proportion of respondents in the lower wealth quintile accessed maternal health utilization services compared to women in the relatively higher wealth quintile.

**Table 2:** Sociodemographic factors and association with maternal health services utilization during pregnancy and birth among using women of reproductive age using the 2022 Ghana DHS (N = 4303).

Variables	Timing of first ANC visit									Completed recommended number of ANC visits									Skilled birth attendance					
	Yes			No						Yes			No						Yes			No		
	n	%		n		%		*p*-value		n		%	n	%			*p*-value		n	%		n	%	*p*-value
**Health insurance coverage**																								
Yes	2271	68.78		1031		31.22		*		3078		93.22	224	6.78			*		2652	80.31		650	19.69	***
No	652	65.13		349		34.87				914		91.31	87	8.69					862	86.11		139	13.89	
**Age**																								
15-25	856	63.13		500		36.87		***		1223		90.19	133	9.81			***		1149	84.73		207	15.27	**
26-34	1325	70.86		545		29.14				1762		94.22	108	5.78					1488	79.57		382	20.43	
35-42	659	70.48		276		29.52				880		94.12	55	5.88					759	81.18		176	18.82	
42-49	83	58.45		59		41.55				127		89.44	15	10.56					118	83.10		24	16.90	
**Marital status**																								
Single	382	62.21		232		37.79		**		546		88.93	68	11.07			***		548	89.25		66	10.75	***
Married	2541	68.88		1148		31.12				3446		93.41	243	6.59					2966	80.40		723	19.60	
**Education**																								
No education	714	65.21		381		34.79		***		993		90.68	102	9.32			***		907	82.83		188	17.17	**
Primary	418	64.41		231		35.59				589		90.76	60	9.24					534	82.28		115	17.72	
Secondary	1459	67.17		713		32.83				2028		93.37	144	6.63					1786	82.23		386	17.77	
Higher	332	85.79		55		14.21				382		98.71	5	1.29					287	74.16		100	25.84	
**Area of residence**																								
Urban	1370	69.26		608		30.74				1861		94.08	117	5.92			**		1598	80.79		380	19.21	
Rural	1553	66.80		772		33.20				2131		91.66	194	8.34					1916	82.41		409	17.59	
**Employment**																								
Unemployed	649	64.51		357		35.49		**		916		91.05	90	8.95			*		821	81.61		185	18.39	
Employed	2274	68.97		1023		31.03				3076		93.30	221	6.70					2693	81.68		604	18.32	
**Religion**																								
No religion	41	57.75		30		42.25		**		60		84.51	11	15.49			*		60	84.51		11	15.49	
Christianity	1924	69.84		831		30.16				2566		93.14	189	6.86					2246	81.52		50.9	18.48	
Islam	922	65.16		493		34.84				1210		92.58	105	7.42					1158	81.84		257	18.16	
Traditional religion	36	58.06		26		41.94				56		90.32	6	9.68					50	80.65		12	19.35	
**Wealth quintile**																								
Lowest	738	63.62		422		36.38		***		1026		88.45	134	11.55			***		964	83.10		196	16.90	***
Second	685	64.68		374		35.32				978		92.35	81	7.65					883	83.38		176	16.62	
Middle	564	67.30		274		32.70				782		93.32	56	6.68					692	82.58		146	17.42	
Fourth	498	71.04		203		28.96				671		95.72	30	4.28					570	81.31		131	18.69	
Highest	438	80.37		107		19.63				535		98.17	10	1.83					405	71.31		140	25.69	
**Parity**																								
Less than 4	1976	69.72		858		30.28		**		2641		93.19	193	6.81			**		2371	83.66		463	16.34	***
More than 3 less than 6	635	65.40		336		31.60				906		93.31	65	6.69					768	79.09		203	20.91	
5 or more	312	62.65		186		37.35				445		89.36	53	10.64					375	75.30		123	24.70	
**Exposure to internet**																								
Not at all	1963	65.22		1047		34.78		***		2752		91.43	258	8.57			***		2476	82.26		534	17.74	
Less than once a week	105	70.00		45		30.00				144		96.00	6	4.00					120	80.00		30	20.00	
At least once a week	262	68.23		122		31.77				361		94.01	23	5.99					314	81.77		70	18.23	
Almost everyday	593	78.13		166		21.87				735		96.84	24	3.16					604	79.58		155	20.42	
**Region**																								
Western	142	75.13		47		24.87		**		183		96.83	6	3.17			***		166	87.83		23	12.17	**
Central	152	69.09		68		30.91				207		94.09	13	5.91					172	78.18		48	21.82	
Greater Accra	144	66.36		73		33.64				205		94.47	12	5.53					171	78.80		46	21.20	
Volta	135	72.97		50		27.03				177		95.68	8	4.32					149	80.54		36	19.46	
Eastern	134	66.67		67		33.33				189		94.03	12	5.97					150	74.63		51	25.37	
Ashanti	195	69.15		87		30.85				262		92.91	20	7.09					229	81.21		53	18.79	
Western North	141	70.85		58		29.15				185		92.96	14	7.04					157	78.89		42	21.11	
Ahafo	166	67.76		79		32.24				228		93.06	17	6.94					208	84.90		37	15.10	
Bono	135	66.50		68		33.50				188		92.61	15	7.39					169	83.25		34	16.75	
Bono East	206	70.07		88		29.93				269		91.50	25	8.50					234	79.59		60	20.41	
Oti	171	72.15		66		27.85				204		86.08	33	13.92					178	75.11		59	24.89	
Northern	215	56.88		163		43.12				349		92.33	29	7.67					318	84.13		60	15.87	
Savannah	214	65.05		115		34.95				294		89.36	35	10.64					266	80.85		63	19.15	
North East	253	63.89		143		36.11				347		87.63	49	12.37					329	83.08		67	16.92	
Upper East	246	71.51		98		28.49				334		97.09	10	2.91					301	87.50		43	12.50	
Upper West	226	73.14		83		26.86				301		97.41	8	2.59					261	84.47		48	15.53	

Variables			Facility-Based Delivery										Post-Natal Care											
			Yes				No						Yes			No								
			n		%		n		%		*p*-value		n		%	n		%			*p*-value			
**Health Insurance Coverage**																								
Yes			2942		89.10		360		10.90		**		3155		95.55	147		4.45			**			
No			930		92.91		71		7.09				930		92.91	71		7.09						
**Age**																								
15-25			1232		90.86		124		9.14				1279		94.32	77		5.68						
26-34			1656		88.72		211		11.28				1783		95.35	87		4.65						
35-42			851		91.02		84		8.98				887		94.87	48		5.13						
42-49			130		91.55		12		8.45				136		95.77	6		4.23						
**Marital Status**																								
Single			586		95.44		28		4.56		***		584		95.11	30		4.86						
Married			3286		89.08		403		1092				3501		94.90	188		5.10						
**Education**																								
No Education			964		88.04		131		11.96				1040		94.98	55		5.02						
Primary			587		90.45		62		9.55				605		93.22	44		6.78						
Secondary			1964		90.42		208		9.58				2070		95.30	102		4.70						
Higher			357		92.25		30		7.75				370		95.61	17		4.39						
**Area Of Residence**																								
Urban			1800		91.00		178		9.00		*		1890		95.55	88		4.45						
Rural			2072		89.12		253		10.88				2195		94.41	130		5.59						
**Employment**																								
Unemployed			886		88.07		120		11.93		*		950		94.43	56		5.57						
Employed			2986		90.57		311		9.43				3135		95.09	162		4.91						
**Religion**																								
No Religion			62		87.32		9		12.68				67		94.37	4		5.63			*			
Christianity			2491		90.42		264		9.58				2637		95.72	118		4.28						
Islam			1263		89.26		152		10.74				1324		93.57	91		6.43						
Traditional Religion			56		90.32		6		9.68				57		91.94	5		8.06						
**Wealth Quintile**																								
Lowest			1026		88.45		134		11.55				1093		94.22	67		5.78			**			
Second			946		89.33		113		10.67				988		93.30	71		6.70						
Middle			761		90.81		77		9.19				799		95.35	39		4.65						
Fourth			639		91.16		62		8.84				678		96.72	23		3.28						
Highest			500		91.74		45		8.26				527		96.70	18		3.30						
**Parity**																								
Less Than 4			2629		92.77		205		7.23		***		2692		94.99	142		5.01						
More Than 3 Less Than 6			830		85.48		141		14.52				927		95.47	44		4.53						
6 or more			413		82.93		85		17.07				466		93.57	32		6.43						
**Exposure To Internet**																								
Not At All			2686		89.24		324		10.76		*		2859		94.98	151		5.02			*			
Less Than once a week			130		86.67		20		13.33				135		90.00	15		10.00						
At Least Once a week			354		92.19		30		7.81				370		96.35	14		3.65						
Almost Everyday			702		92.46		57		7.51				721		94.99	38		5.01						
**Region**																								
Western			170		89.95		19		10.05				188		99.47	1		0.53			**			
Central			193		87.73		27		12.27				209		95.00	11		5.00						
Greater Accra			201		92.63		16		7.37				216		99.54	1		0.46						
Volta			169		91.35		16		8.65				171		92.43	14		7.57						
Eastern			186		92.54		15		7.46				199		99.00	2		1.00						
Ashanti			257		91.13		25		8.87				262		92.91	20		7.09						
Western North			173		86.93		26		13.07				190		95.48	9		4.52						
Ahafo			219		89.39		26		10.61				235		95.92	10		4.08						
Bono			184		90.64		19		9.36				197		97.04	6		2.96						
Bono East			265		90.14		29		9.86				288		97.96	6		2.04						
Oti			207		87.34		30		12.66				232		97.89	5		2.11						
Northern			347		91.80		31		8.20				346		91.35	32		8.47						
Savannah			290		88.15		39		11.85				305		92.71	24		7.29						
North East			347		87.63		49		12.37				359		90.66	37		9.34						
Upper East			313		90.99		31		9.01				330		95.93	14		4.07						
Upper West			285		92.23		24		7.77				286		92.56	23		7.44						

*p*  <  0.05, *p*  <  0.01 and *p*  <  0.001 indicated by *, ** and *** respectively.

### Predictors of health insurance coverage among women of reproductive age in Ghana

Respondents having higher education (aOR 3.01; 1.86–4.85), identifying as a Muslim (aOR 2.60; 1.57–4.31) belonging to the fourth (aOR 1.49; 1.10–2.03) and highest (aOR 1.65; 1.12–2.44) wealth quintile were found to be more likely to be insured. Also, other predictors of health insurance coverage among women of reproductive age were residing in the Eastern (aOR 4.22; 2.38–7.50), Western North (aOR 2.34; 1.43–3.83), Ahafo ((aOR 1.94; 1.23–3.07), Bono (aOR 1.88; 1.17–3.02), Bono East (aOR 2.00; 1.29–3.12), Oti (aOR; 1.10-2.03-3.09), North East (aOR 1.67; 1.08–2.58) and Upper East (aOR 1.78; 1.15–2.75). Respondents who are employed (aOR 0.59; 0.49–0.72) were less likely to be enrolled in the health insurance scheme (*p*-value < 0.001) as shown in Table 3.

**Table 3:** Adjusted Odds ratio for association between socio-demographic predictors and health insurance coverage among women of reproductive age using the 2022 Ghana DHS (N = 4303).

Covariates	Health insurance coverage (Yes)			
	aOR	95% CI		*p*-value
		Lower	Higher	
**Age**				
15-24	1.00			
25-34	1.04	0.86	1.27	
35-42	0.87	0.66	1.13	
42-49	1.06	0.66	1.69	
**Marital status**				
Single	1.00			
Married	1.23	0.99	1.54	
**Education**				
No education	1.00			
Primary	0.97	0.77	1.23	
Secondary	1.14	0.93	1.42	
Higher	3.01	1.86	4.85	***
**Residence**				
Urban	1.00			
Rural	1.12	0.93	1.35	
**Employment**				
Unemployed	1.00			
Employed	0.59	0.49	0.72	***
**Religion**				
No religion	1.00			
Christianity	2.00	1.22	3.28	**
Islam	2.60	1.57	4.31	**
Traditional religion	0.95	0.47	1.92	
**Wealth quintile**				
Lowest	1.00			
Second	1.16	0.94	1.43	
Middle	1.06	0.82	1.36	
Fourth	1.49	1.10	2.03	*
Highest	1.65	1.12	2.44	*
**Parity**				
Less than 4	1.00			
More than 3 less than 6	0.98	0.79	1.22	
6 or more	0.94	0.70	1.26	
**Internet exposure**				
Not at all	1.00			
Less than once a week	0.89	0.60	1.33	
At least once a week	1.30	0.97	1.75	
Almost everyday	1.28	0.98	1.67	
**Region**				
Western	1.00			
Central	1.09	0.71	1.69	
Greater Accra	1.12	0.71	1.77	
Volta	1.44	0.90	2.31	
Eastern	4.22	2.38	7.50	***
Ashanti	1.47	0.95	2.26	
Western North	2.34	1.43	3.83	**
Ahafo	1.94	1.23	3.07	**
Bono	1.88	1.17	3.02	*
Bono East	2.00	1.29	3.12	**
Oti	1.96	1.24	3.09	**
Northern	1.52	0.97	2.36	
Savannah	0.81	0.53	1.24	
North East	1.67	1.08	2.58	*
Upper East	1.78	1.15	2.75	**
Upper West	1.51	0.97	2.36	

*p *< 0.05, *p* < 0.01 and *p *< 0.001 indicated by *, ** and *** respectively.

### Association between health insurance and maternal health services utilization

Multivariate logistic regression to determine the association between health insurance coverage and maternal health insurance utilization adjusted for socio- demographic factors is shown in Table 4. Health insurance coverage is associated with the likely odds of post-natal care (aOR 1.56; 1.15–2.12). Counterintuitively, respondents with health insurance coverage showed reduced odds of having skilled birth attendance (aOR 0.7; 0.57–0.86) and facility-based delivery (aOR 0.59; 0.45–0.78). The likelihood of an early first ANC visit had a significant association with women aged 25–34 (aOR 1.34; 1.13–1.60), 35–42 (aOR 163; 1.28–2.09), higher-educated women (aOR 1.50; 1.02–2.21), and women of the highest wealth quintile (aOR 1.67; 1.18–2.37). Women with 3 or less children are more likely to attend their first ANC visit in the first trimester compared to those with 4 or more children (aOR 0.7; 0.57–0.84) and 6 or more (aOR 0.6; 0.46–0.80).Being married is a predictor for the likely utilization of maternal health services: Timing of first ANC visit (aOR 1.31; 1.08–1.60) and completing the recommended number of ANC visits (aOR 1.57; 1.13–2.17). However, there are reduced odds for when a woman is married to have a facility-based delivery (aOR 0.40; 0.26–0.60) or skilled birth attendance (aOR 0.50; 0.38–0.67) and were statistically significant with p-values of <0.001 and 0.001 respectively

**Table 4:** Adjusted Odds ratio for association health insurance coverage between and maternal health utilization among women of reproductive age using the 2022 Ghana DHS (N = 4303).

Variable	Timing of first ANC visit				Completed recommended number of ANC visits					Skilled birth attendance			
	aOR	95% CI		*p*-value	aOR	95% CI		*p*-value		aOR	95% CI		*p*-value
			Lower	Upper		Lower	Upper	Lower		Upper			
**Health insurance coverage**													
No	1.00				1.00					1.00			
Yes	1.08	0.922	1.26		1.12	0.86	1.47			0.70	0.57	0.86	**
**Covariates**													
**Age**													
15-24	1.00				1.00					1.00			
25-34	1.34	1.13	1.60	**	1.54	1.11	2.12	**		1.04	0.84	1.30	
35-42	1.63	1.28	2.09	***	2.03	1.26	3.25	**		1.58	1.18	2.13	**
42-49	1.16	0.77	1.76		1.51	0.74	3.07			2.19	1.29	3.74	**
**Marital status**													
Single	1.00				1.00					1.00			
Married	1.31	1.08	1.60	**	1.57	1.13	2.17	**		0.50	0.38	0.67	***
**Education**													
No education	1.00				1.00					1.00			
Primary	0.84	0.67	1.04		0.89	0.61	1.28			0.91	0.69	1.20	
Secondary	0.84	0.69	1.02		1.01	0.72	1.42			0.82	0.64	1.04	
Higher	1.50	1.02	2.21	*	1.83	0.65	5.14			0.55	0.37	0.81	**
**Area of residence**													
Urban	1.00				1.00					1.00			
Rural	1.11	0.94	1.31		1.15	0.85	1.56			1.02	0.84	1.25	
**Employment**													
Unemployed	1.00				1.00					1.00			
Employed	1.11	0.95	1.30		1.20	0.91	1.57			1.12	0.93	1.37	
**Religion**													
No religion	1.00				1.00					1.00			
Christianity	1.44	0.88	2.36		1.70	0.85	3.40			0.88	0.45	1.72	
Islam	1.30	0.79	2.15		1.80	0.89	3.66			0.87	0.44	1.72	
Traditional religion	1.19	0.58	2.43		2.00	0.67	5.97			0.81	0.32	2.04	
**Wealth quintile**													
Lowest	1.00				1.00					1.00			
Second	1.06	0.88	1.29		1.68	1.22	2.32	**		1.09	0.86	1.39	
Middle	1.18	0.94	1.49		1.88	1.25	2.83	**		1.08	0.81	1.44	
Fourth	1.30	0.99	1.71		2.55	1.51	4.32	**		0.97	0.70	1.35	
Highest	1.67	1.18	2.37	**	4.27	1.90	9.59	***		0.64	0.43	0.94	*
**Parity**													
Less than 4	1.00				1.00					1.00			
More than 3 less than 6	0.7	0.57	0.84	***	0.79	0.54	1.14			0.59	0.47	0.73	***
6 or more	0.6	0.46	0.80	***	0.51	0.32	0.83	**		0.36	0.26	0.49	***
**Exposure to internet**													
Not at all	1.00				1.00					1.00			
Less than once a week	1.24	0.86	1.81		1.74	0.75	4.07			0.82	0.53	1.26	
At least once a week	0.97	0.76	1.25		0.95	0.59	1.52			1.05	0.78	1.42	
Almost everyday	1.30	1.03	1.63	*	1.37	0.84	2.24			1.19	0.91	1.57	
**Region**													
Western	1.00				1.00					1.00			
Central	0.77	0.50	1.21		0.54	0.20	1.46			0.42	0.24	0.73	**
Greater Accra	0.52	0.33	0.81	**	0.39	0.14	1.07			0.50	0.29	0.88	*
Volta	0.87	0.54	1.40		0.72	0.24	2.13			0.53	0.29	0.94	*
Eastern	0.63	0.40	0.99	*	0.49	0.18	1.35			0.38	0.22	0.65	**
Ashanti	0.76	0.50	1.17		0.47	0.18	1.22			0.56	0.32	0.95	*
Western North	0.87	0.55	1.38		0.52	0.19	1.42			0.47	0.27	0.83	**
Ahafo	0.83	0.53	1.28		0.64	0.24	1.70			0.71	0.40	1.26	
Bono	0.68	0.43	1.06		0.46	0.17	1.24			0.61	0.34	1.09	
Bono East	0.93	0.60	1.43		0.51	0.20	1.29			0.46	0.27	0.79	**
Oti	1.00	0.64	1.58		0.28	0.11	0.69	0.006		0.36	0.21	0.62	**
Northern	0.47	0.31	0.72	**	0.48	0.19	1.25	0.132		0.72	0.41	1.27	
Savannah	0.78	0.51	1.21		0.43	0.17	1.09	0.075		0.48	0.28	0.84	*
North East	0.73	0.47	1.12		0.38	0.15	0.96	0.040		0.64	0.37	1.11	
Upper East	0.90	0.58	1.37		1.55	0.54	4.48	0.415		0.88	0.50	1.55	
Upper West	0.98	0.63	1.53		1.62	0.53	4.89	0.396		0.71	0.41	1.25	

Variables		Facility-based delivery					Post-natal care						
		aOR	95% CI		*p*-value		aOR		95% CI		*p*-value		
			Lower	Upper					Lower	Upper			
**Health insurance coverage**													
No		1.00					1.00						
Yes		0.59	0.45	0.78	**		1.56		1.15	2.12	**		
**Covariates**													
**Age**													
15-24		1.00					1.00						
25-34		1.51	1.12	2.03	**		1.06		0.73	1.54			
35-42		3.69	2.45	5.55	***		1.00		0.59	1.71			
42-49		5.61	2.76	11.41	***		1.38		0.52	3.67			
**Marital status**													
Single		1.00					1.00						
Married		0.40	0.26	0.60	***		1.10		0.71	1.73			
**Education**													
No education		1.00					1.00						
Primary		1.13	0.80	1.60			0.49		0.32	0.77	**		
Secondary		0.86	0.64	1.16			0.64		0.42	0.97	*		
Higher		0.71	0.40	1.25			0.69		0.32	1.48			
**Area of residence**													
Urban		1.00					1.00						
Rural		1.00	0.77	1.29			0.87		0.61	1.24			
**Employment**													
Unemployed		1.00					1.00						
Employed		1.41	1.11	1.80	**		1.07		0.76	1.49			
**Religion**													
No religion		1.00					1.00						
Christianity		1.25	0.60	2.63			1.19		0.41	3.41			
Islam		1.24	0.58	2.62			1.14		0.39	3.28			
Traditional religion		1.43	0.46	4.47			0.78		0.19	3.17			
**Wealth quintile**													
Lowest		1.00					1.00						
Second		1.07	0.80	1.43			0.78		0.53	1.15			
Middle		1.25	0.86	1.81			1.02		0.62	1.70			
Fourth		1.17	0.76	1.79			1.36		0.73	2.54			
Highest		1.02	0.60	1.75			1.32		0.61	2.87			
**Parity**													
Less than 4		1.00					1.00						
More than 3 less than 6		0.28	0.21	0.38	***		1.05		0.68	1.61			
6 or more		0.15	0.10	0.23	***		0.69		0.39	1.22			
**Exposure to internet**													
Not at all		1.00					1.00						
Less than once a week		0.61	0.36	1.02			0.42		0.23	0.76	**		
At least once a week		1.15	0.75	1.76			1.03		0.57	1.88			
Almost everyday		1.22	0.84	1.79			0.58		0.36	0.93	*		
**Region**													
Western		1.00					1.00						
Central		0.69	0.36	1.32			0.10		0.01	0.82	*		
Greater Accra		1.09	0.53	2.23			0.98		0.06	15.93			
Volta		1.11	0.54	2.28			0.07		0.01	0.50	**		
Eastern		1.35	0.65	2.81			0.50		0.05	5.59			
Ashanti		1.08	0.57	2.08			0.07		0.01	0.54	*		
Western North		0.77	0.40	1.49			0.11		0.01	0.87	*		
Ahafo		1.04	0.54	2.01			0.13		0.02	1.05			
Bono		0.98	0.49	1.97			0.17		0.02	1.42			
Bono East		1.11	0.58	2.12			0.26		0.03	2.19			
Oti		0.82	0.43	1.56			0.26		0.03	2.30			
Northern		1.55	0.80	3.02			0.05		0.01	0.41	**		
Savannah		0.87	0.46	1.65			0.07		0.01	0.52	*		
North East		1.07	0.57	2.02			0.04		0.01	0.34	**		
Upper East		1.18	0.62	2.25			0.12		0.02	0.95	*		
Upper West		1.54	0.78	3.03			0.06		0.01	0.48	**		

*p*  <  0.05, *p*  <  0.01 and *p*  <  0.001 indicated by *, ** and *** respectively.

### Subgroup analysis of ROC

The performance of the logistic regression model in predicting the outcome was evaluated using the area under the Receiver Operating Characteristic (ROC) curve (AUC). For completing the recommended number of ANC visits and post-natal care subgroups, the model demonstrated an AUC of 0.72 (0.69 to 0.75) and 0.72 (0.69 to 0.75) respectively, indicating acceptable discriminatory ability. All the other models returned AUC within the acceptable discriminatory as indicated in S1 Fig. As a reference, an AUC of 0.5 corresponds to a model with no discriminatory ability, while the observed AUC are all between 0.62 and 0.72 suggests that the model performs better than random chance. These statistics further support the models’ moderate ability to differentiate between the outcome categories within each subgroup.

## Discussion

This paper aimed to investigate the association between health insurance coverage and maternal health services utilization among women of reproductive age in Ghana by using timing of ANC first visit, completion of ANC visits during pregnancy, skilled birth attendance, facility-based delivery, and post-natal care as indicators. The study also investigated health insurance enrollment across different socio-demographic categorizations using the most recent national survey data. Women enrolled in the NHIS in Ghana are eligible for free maternal health services from all NHIS-accredited health facilities under the free maternal care policy. It is reported that, as of 2021, the NHIS had about 15 million active members registered with the scheme which leaves almost 50% of the population uninsured [21].

In this study, 76.74% of the respondents reported having registered with the NHIS. This finding is comparable to the other studies though this study reports a slightly higher proportion of respondents having health insurance coverage [16,22]. This seems to show a steady increase in the enrollment in the health insurance scheme by women compared to previous years where in 2018 about 40% of participants were reported to have insurance [23]. Maternal health utilization from the findings of this study is associated with having insurance. This could be because of the benefits of the free maternal care policy when registered with the scheme. The policy covers a wide range of services from ANC through to post-natal care. Uninsured women seeking maternal care services are required to pay out-of-pocket for all services. Similar findings were observed in a study done in Tanzania where health insurance coverage was associated with maternal health service utilization [24].This finding also confirms studies done in Ghana but on a much smaller scale [16,25,26].Findings in this study suggest that age, education level, employment status, religion, wealth quintile, parity, exposure to the internet and region of residence are associated with the timing of first ANC visit and completion of the recommended number of ANC visits. Other studies have determined similar observations of associations between timing of first ANC and these above-listed socio-demographic characteristics [27]. Apart from post-natal care, marital status is associated with all other maternal health service indicators. The education level of respondents was not a predictor of having skilled birth attendance, facility-based delivery or post-natal care, which was an unexpected finding. The outcome maybe be due to the study population and further in-depth studies may be required to unravel the attributable factors for the outcome.

The findings of this study revealed that having health insurance coverage did increase the odds of initiating timing of ANC visit on time or completing the recommended number of ANC visits there was no statistical difference. This finding was similar to other studies where health insurance coverage did not statistically increase the odds of initiating ANC on time and completion of ANC visits [23,28]. However, the finding is at variance with publications of studies from other sub-Saharan African countries and Asian countries where there was a statistical difference between insured and uninsured women with regards to initiation of ANC and completion of the recommended ANC visits [29–31]. However, post-natal care was influenced by having health insurance coverage. Curiously, the finding of this study found there were decreased odds of insured women having skilled birth attendance and facility-based delivery. the reasons for this may require further investigation, however, the implementation health insurance scheme in Ghana has been fraught with many challenges. More often than not, health facilities still demand out-of-pocket payments for services supposedly covered by the health insurance due to inadequate funding mechanisms of the health insurance and subsequent late reimbursement of providers [32–36]. This may account for the dissatisfaction with service provision under the health insurance and resulting in abstinence from services utilization by insured women. This assertion could be buttressed by the finding that women within the highest wealth quintile were 67% more likely to initiate ANC on time and 327% more likely to complete the recommended number of ANC visits compared to the women in the lowest quintile, however, the highest wealth quintile was proportionally the lowest insured category. This seems to suggest that the most insured population, which is the women in the lowest wealth quintile are not utilizing some maternal health services as expected pointing to some level of dissatisfaction with the service provision under the insurance policy.

Socio-demographic factors like marital status, age, and employment increase the likelihood of facility-based birth among respondents of this study. This finding is concurrent with other studies conducted in other low-and-middle-income countries [37,38].

This study utilized the data from the latest national survey and is reflective of the association between health insurance coverage and maternal health utilization among women of reproductive age in Ghana. However, this study has some limitations. First, this study used cross-sectional data and as such the findings cannot infer causality. Second, the survey questions about health insurance enrollment were administered to women not at the time of pregnancy or delivery but at the time of the interview. Third, this study however narrowed out the sample size to women who have had previous pregnancies within the past five years of before the study. Despite these limitations, this study provides evidence of the associations between the utilization of maternal health services and health insurance coverage among women of reproductive age in Ghana based on the latest nationally representative data.

## Conclusion

Evidence from this study reveals that even though health insurance coverage may increase the likelihood of maternal health services utilization, the implementation practices of these policies may have a more profound impact. This study also revealed the influence of other socio-demographic factors on health insurance enrollment and maternal health service utilization. Challenges faced by the health insurance policy like out-of-pocket payments while insured should be addressed to further improve the utilization of health services covered under the policy.

## Supporting information

S1 File Multicollinearity Assessment, Frequency distribution of outcome variables, Variable List and ROC analysis. (PDF)
