# Correction: Health insurance enrollment and maternal health service utilization using Ghana Demographic and Health Survey, 2022

**DOI:** 10.1371/journal.pone.0352273

**Published:** 2026-06-23

**Authors:** The PLOS One Staff

In Table 4, the subheadings Lower and Upper have shifted right. As a result, there are mislabeled columns in Table 4. Please see the correct Table 4 here.

The publisher apologizes for the errors.

**Table 4:** Adjusted Odds ratio for association between health insurance coverage and maternal health utilization among women of reproductive age using the 2022 Ghana DHS (N=4303).

Variables	Timing of first ANC visit				Completed recommended number of ANC visits				Skilled Birth Attendance			
	aOR	95%CI		*p-value*	aOR	95%CI		*p-value*	aOR	95%CI		*p-value*
		LOWER	UPPER			LOWER	UPPER			LOWER	UPPER	
Health Insurance Coverage												
No	1.00				1.00				1.00			
Yes	1.08	0.922	1.26		1.12	0.86	1.47		0.70	0.57	0.86	**
Covariates												
Age												
15-24	1.00				1.00				1.00			
25-34	1.34	1.13	1.60	**	1.54	1.11	2.12	**	1.04	0.84	1.30	
35-42	1.63	1.28	2.09	***	2.03	1.26	3.25	**	1.58	1.18	2.13	**
42-49	1.16	0.77	1.76		1.51	0.74	3.07		2.19	1.29	3.74	**
Marital Status												
Single	1.00				1.00				1.00			
Married	1.31	1.08	1.60	**	1.57	1.13	2.17	**	0.50	0.38	0.67	***
Education												
No Education	1.00				1.00				1.00			
Primary	0.84	0.67	1.04		0.89	0.61	1.28		0.91	0.69	1.20	
Secondary	0.84	0.69	1.02		1.01	0.72	1.42		0.82	0.64	1.04	
Higher	1.50	1.02	2.21	*	1.83	0.65	5.14		0.55	0.37	0.81	**
Area of residence												
Urban	1.00				1.00				1.00			
Rural	1.11	0.94	1.31		1.15	0.85	1.56		1.02	0.84	1.25	
Employment												
Unemployed	1.00											
Employed	1.11	0.95	1.30		1.20	0.91	1.57		1.12	0.93	1.37	
Religion												
No Religion	1.00				1.00				1.00			
Christianity	1.44	0.88	2.36		1.70	0.85	3.40		0.88	0.45	1.72	
Islam	1.30	0.79	2.15		1.80	0.89	3.66		0.87	0.44	1.72	
Traditional Religion	1.19	0.58	2.43		2.00	0.67	5.97		0.81	0.32	2.04	
Wealth Quintile												
Lowest	1.00				1.00				1.00			
Second	1.06	0.88	1.29		1.68	1.22	2.32	**	1.09	0.86	1.39	
Middle	1.18	0.94	1.49		1.88	1.25	2.83	**	1.08	0.81	1.44	
Fourth	1.30	0.99	1.71		2.55	1.51	4.32	**	0.97	0.70	1.35	
Highest	1.67	1.18	2.37	**	4.27	1.90	9.59	***	0.64	0.43	0.94	*
Parity												
Less than 4	1.00				1.00				1.00			
More than 3 less than 6	0.7	0.57	0.84	***	0.79	0.54	1.14		0.59	0.47	0.73	***
More than 5	0.6	0.46	0.80	***	0.51	0.32	0.83		0.36	0.26	0.49	***
Exposure to Internet												
Not at all	1.00				1.00				1.00			
Less than once a week	1.24	0.86	1.81		1.74	0.75	4.07		0.82	0.53	1.26	
At least once a week	0.97	0.76	1.25		0.95	0.59	1.52		1.05	0.78	1.42	
Almost everyday	1.30	1.03	1.63	*	1.37	0.84	2.24		1.19	0.91	1.57	
Region												
Western	1.00				1.00				1.00			
Central	0.77	0.50	1.21		0.54	0.20	1.46		0.42	0.24	0.73	**
Greater Accra	0.52	0.33	0.81	**	0.39	0.14	1.07		0.50	0.29	0.88	*
Volta	0.87	0.54	1.40		0.72	0.24	2.13		0.53	0.29	0.94	*
Eastern	0.63	0.40	0.99	*	0.49	0.18	1.35		0.38	0.22	0.65	**
Ashanti	0.76	0.50	1.17		0.47	0.18	1.22		0.56	0.32	0.95	*
Western North	0.87	0.55	1.38		0.52	0.19	1.42		0.47	0.27	0.83	**
Ahafo	0.83	0.53	1.28		0.64	0.24	1.70		0.71	0.40	1.26	
Bono	0.68	0.43	1.06		0.46	0.17	1.24		0.61	0.34	1.09	
Bono East	0.93	0.60	1.43		0.51	0.20	1.29		0.46	0.27	0.79	**
Oti	1.00	0.64	1.58		0.28	0.11	0.69	*	0.36	0.21	0.62	**
Northern	0.47	0.31	0.72	**	0.48	0.19	1.25		0.72	0.41	1.27	
Savannah	0.78	0.51	1.21		0.43	0.17	1.09		0.48	0.28	0.84	*
North East	0.73	0.47	1.12		0.38	0.15	0.96	*	0.64	0.37	1.11	
Upper East	0.90	0.58	1.37		1.55	0.54	4.48		0.88	0.50	1.55	
Upper West	0.98	0.63	1.53		1.62	0.53	4.89		0.71	0.41	1.25	
**Variables**	**Facility-based Delivery**				**Post-natal Care**			
	**aOR**	**95%CI**		***p-value***	**aOR**	**95%CI**		***p-value***
		**LOWER**	**UPPER**			**LOWER**	**UPPER**	
**Health Insurance Coverage**								
No	1.00				1.00			
Yes	0.59	0.45	0.78	**	1.56	1.15	2.12	**
**Covariates**								
**Age**								
15-24	1.00				1.00			
25-34	1.51	1.12	2.03	**	1.06	0.73	1.54	
35-42	3.69	2.45	5.55	***	1.00	0.59	1.71	
42-49	5.61	2.76	11.41	***	1.38	0.52	3.67	
**Marital Status**								
Single	1.00				1.00			
Married	0.40	0.26	0.60	***	1.10	0.71	1.73	
**Education**								
No Education	1.00				1.00			
Primary	1.13	0.80	1.60		0.49	0.32	0.77	**
Secondary	0.86	0.64	1.16		0.64	0.42	0.97	*
Higher	0.71	0.40	1.25		0.69	0.32	1.48	
**Area of residence**								
Urban	1.00				1.00			
Rural	1.00	0.77	1.29		0.87	0.61	1.24	
**Employment**								
Unemployed	1.00				1.00			
Employed	1.41	1.11	1.80	**	1.07	0.76	1.49	
**Religion**								
No Religion	1.00				1.00			
Christianity	1.25	0.60	2.63		1.19	0.41	3.41	
Islam	1.24	0.58	2.62		1.14	0.39	3.28	
Traditional Religion	1.43	0.46	4.47		0.78	0.19	3.17	
**Wealth Quintile**								
Lowest	1.00				1.00			
Second	1.07	0.80	1.43		0.78	0.53	1.15	
Middle	1.25	0.86	1.81		1.02	0.62	1.70	
Fourth	1.17	0.76	1.79		1.36	0.73	2.54	
Highest	1.02	0.60	1.75		1.32	0.61	2.87	
**Parity**								
Less than 4	1.00				1.00			
More than 3 less than 6	0.28	0.21	0.38	***	1.05	0.68	1.61	
More than 5	0.15	0.10	0.23	***	0.69	0.39	1.22	
**Exposure to Internet**								
Not at all	1.00				1.00			
Less than once a week	0.61	0.36	1.02		0.42	0.23	0.76	**
At least once a week	1.15	0.75	1.76		1.03	0.57	1.88	
Almost everyday	1.22	0.84	1.79		0.58	0.36	0.93	*
**Region**								
Western	1.00				1.00			
Central	0.69	0.36	1.32		0.10	0.01	0.82	*
Greater Accra	1.09	0.53	2.23		0.98	0.06	15.93	
Volta	1.11	0.54	2.28		0.07	0.01	0.50	**
Eastern	1.35	0.65	2.81		0.50	0.05	5.59	
Ashanti	1.08	0.57	2.08		0.07	0.01	0.54	
Western North	0.77	0.40	1.49		0.11	0.01	0.87	
Ahafo	1.04	0.54	2.01		0.13	0.02	1.05	
Bono	0.98	0.49	1.97		0.17	0.02	1.42	
Bono East	1.11	0.58	2.12		0.26	0.03	2.19	
Oti	0.82	0.43	1.56		0.26	0.03	2.30	
Northern	1.55	0.80	3.02		0.05	0.01	0.41	**
Savannah	0.87	0.46	1.65		0.07	0.01	0.52	*
North East	1.07	0.57	2.02		0.04	0.01	0.34	**
Upper East	1.18	0.62	2.25		0.12	0.02	0.95	*
Upper West	1.54	0.78	3.03		0.06	0.01	0.48	**

p<0.05, p<0.01 and p<0.001 indicated by *, ** and *** respectively.
