# Supplementary material for: Health insurance enrollment and maternal health service utilization using Ghana Demographic and Health Survey, 2022
Source: PLoS One. 2025 Jun 26;20(6):e0325240. doi: 10.1371/journal.pone.0325240 (PMC12200674; doi:10.1371/journal.pone.0325240)

Table S1 Variance Inflation Factor (VIF) Analysis for Assessing Multicollinearity Among Independent Variables

Variable	Parameter Estimate	Standard Error	Variance Inflation
Health Insurance Coverage	0.00966	0.00938	1.02808
Age	0.01804	0.00642	1.79275
Education	0.00597	0.00524	1.67821
Residence	0.01387	0.00952	1.47367
Marital Status	0.04074	0.01174	1.10402
Employment	0.01266	0.00953	1.06591
Wealth Quintile	0.01933	0.00417	2.121
Religion	0.00806	0.00772	1.12026
Parity	-0.02144	0.00776	1.89375
Exposure To Internet	0.00333	0.00401	1.507
Region	0.00011	0.00032	1.03891

Table S2 Frequency distribution of outcome variables (N=4303)

**Variable**	n	%
**Timing of first ANC visit**		
Yes	2923	67.93
No	1380	32.07
**Completed recommended number of ANC visits**		
Yes	3992	92.77
No	311	7.23
**Skilled birth attendance**		
Yes	3514	81.66
No	789	18.34
**Facility-based delivery**		
Yes	3872	89.98
No	431	10.02
**Post-natal care**		
Yes	4085	94.93
No	218	5.07

Table S3 List of Variables

Variables	Type	Measurement	Original Variable
**Dependent Variables**			
Timing of first ANC visit	Binary	0 No<br>1 Yes	*M13*
Completed recommended number of ANC Visits	Binary	0 No<br>1 Yes	*M14*
Facility-based delivery	Binary	0 No<br>1 Yes	*M15*
Skilled Birth Attendance	Binary	0 No<br>1 Yes	*M3A-N*
Post-Natal Care	Binary	0 No<br>1 Yes	*M62*
**Independent Variables**			
Age	Categorical	1 15-24<br>2 25-34<br>3 35-42<br>4 42-49	*V012*
NHIS	Binary	0 No<br>1 Yes	*S1119*
Parity	Categorical	1 less than 4<br>2 More than 3 less than 6<br>3 6 or more	*V201*
Marital status	Categorical	0 Single, divorced, widowed<br>1 Married<br>2 Cohabitation	*V501*
Education Level	Categorical	0 No education<br>1 Primary<br>2 Secondary<br>3 Tertiary	*V106*
Residence	Binary	1 Urban<br>2 Rural	*V025*
Wealth Index	Categorial	1 Poorest<br>2 Poorer<br>3 Middle<br>4 Richer<br>5 Richest	*V190*
Religion	Categorical	0 No religion<br>1 Christianity<br>2 Islam<br>3 Traditional	*V130*
Media Exposure (Internet)	Categorical	0 Not at all<br>1 Less than once a week<br>2 At least once a week<br>3 Almost every day	*V171B*
Region	Categorical	1 Western<br>2 Central	*V024*

	3 Greater Accra<br>4 Volta<br>5 Eastern<br>6 Ashanti<br>7 Western North<br>8 Ahafo<br>9 Bono<br>10 Bono East<br>11 Oti<br>12 Northern<br>13 Savannah<br>14 North East<br>15 Upper East<br>16 Upper West

Figure S1 ROC for logistic regression

Timing of 1st ANC visit

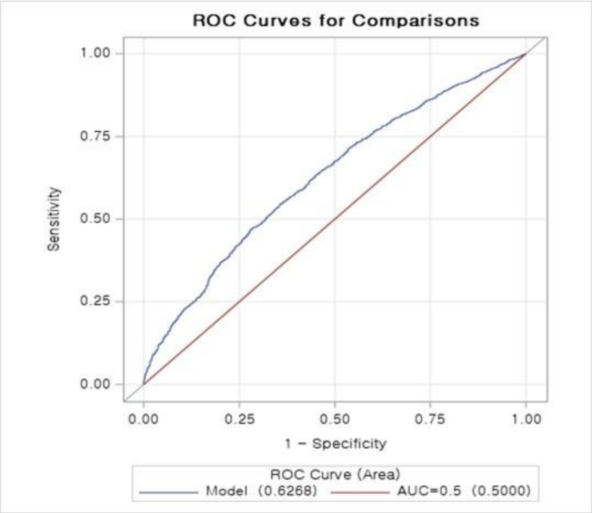

Total number of ANC

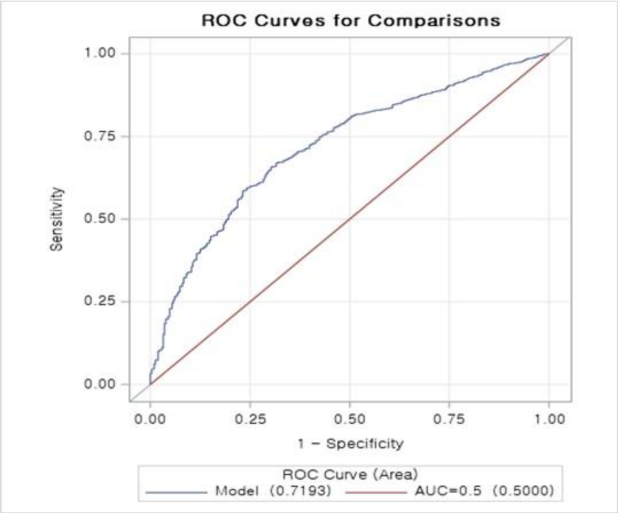

Skilled birth attendance

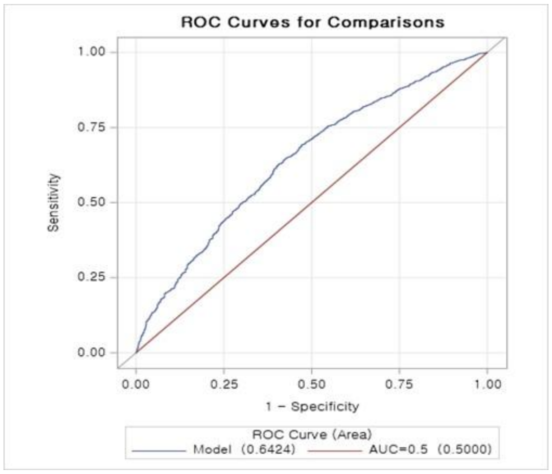

Facility-based delivery

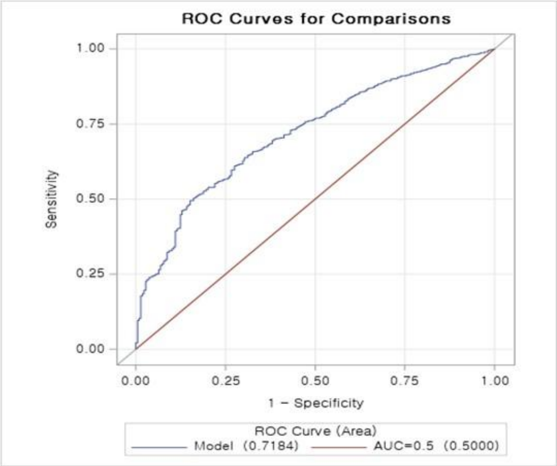

Post-natal care

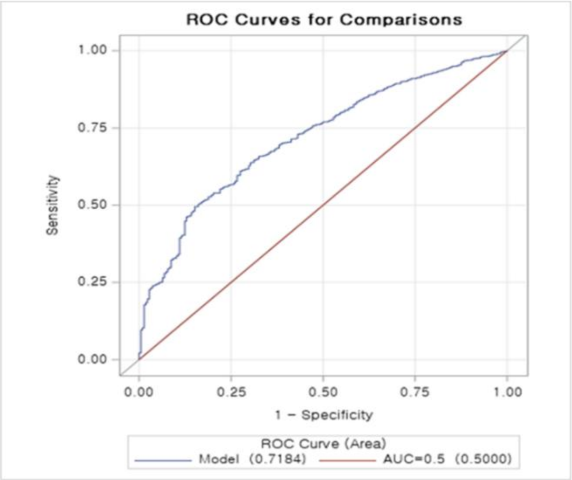

Health Insurance Coverage

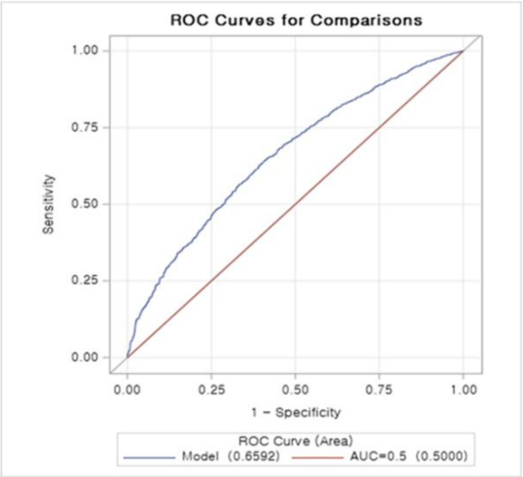

Supplement: S1 File — (PDF) [file pone.0325240.s001.pdf]
